# WHAT IS MORE DANGEROUS- SUICIDALITY IN EPILEPSY OR EPILEPSY AS A DISEASE? _A REVIEW

**DOI:** 10.1192/j.eurpsy.2023.1942

**Published:** 2023-07-19

**Authors:** H. Arshad, K. Hussain, M. Khalid, F. Arain, A. R. Khan, A. Arshad

**Affiliations:** 1 Psychiatry, Jinnah Sindh medical university, Karachi; 2 Medicine, Aziz Bhatti Shaheed Teaching Hospital, Gujrat; 3 Psychiatry, Allama Iqbal Medical College, lahore, Pakistan; 4 Psychiatry, Rutgers New Jersey School of Medicine, New Jersey; 5 Psychiatry, Carilion Clinic Virginia Tech, Virginia, United States

## Abstract

**Introduction:**

Epilepsy is a neurologic condition characterized by spontaneous jerky body movements. It is a chronic morbid condition mostly diagnosed during childhood. Patients are maintained on long-term medications to prevent recurrent seizures that can damage the brain. Medications used for the management of epilepsy have several side effects and require proper monitoring. Patients with epilepsy are at increased risk of psychiatric comorbidities.

**Objectives:**

Our aim is to find factors responsible for causing suicidality in patients with epilepsy.

**Methods:**

A review was conducted using Pubmed database with the search terms [epilepsy] OR [neurological conditions] OR [suicide] OR [suidical attempt] OR [suicidal ideation] OR [depression] OR [psychiatric diseases] OR [mood disorders] OR [anxiety] OR [sleeplessness] which yielded around 800 articles. The number was later reduced to be centered around the main area of interest and produced around 40 articles.

**Results:**

Results show that many contributing factors play an unavoidable role in promoting suicidal ideation that can lead to suicidal attempts in epilepsy. The stigma associated with epilepsy leads to social isolation, lack of opportunities, financial constraints, and impact on close relationships which can be the reasons for depression. According to the review, the foremost contributing factor is the underlying social, emotional, and economic condition of epilepsy patients. Depression and anxiety are the most prevalent psychiatric comorbidities in epileptic patients. Epileptic patients who develop psychiatric ailments show decreased adherence to medications that further worsen the problem.

**Conclusions:**

Though, this area in neurology has started getting attention for further research and guidelines. But efforts are still inadequate for this to be put into clinical practice. More desperate actions needed to be taken for proper diagnosis and management of suicidal ideations by proper use of assessment tools so that timely actions are planned. This is a highly demanding area due to the impact of depressive symptoms on the prognosis of the chronic neurologic condition.

Keywords: Suicidality; Epilepsy; neurology.

**Disclosure of Interest:**

None Declared

